# RMRP accelerates ligamentum flavum hypertrophy by regulating GSDMD-mediated pyroptosis through Gli1 SUMOylation

**DOI:** 10.3389/fimmu.2024.1427970

**Published:** 2024-08-16

**Authors:** Xudong Yan, Tinglong Liu, Run Zhang, Qinghong Ma, Chao Sun

**Affiliations:** Department of Spine Surgery, The Affiliated Jiangning Hospital of Nanjing Medical University, Nanjing, Jiangsu, China

**Keywords:** ligamentum flavum, RMRP, GLI1, GSDMD, hypertrophy, fibrosis

## Abstract

Hypertrophy of ligamentum flavum (LF) is a significant contributing factor to lumbar spinal canal stenosis (LSCS). lncRNA plays a vital role in organ fibrosis, but its role in LF fibrosis remains unclear. Our previous findings have demonstrated that Hedgehog-Gli1 signaling is a critical driver leading to LF hypertrophy. Through the RIP experiment, our group found lnc-RMRP was physically associated with Gli1 and exhibited enrichment in Gli1-activated LF cells. Histological studies revealed elevated expression of RMRP in hypertrophic LF. *In vitro* experiments further confirmed that RMRP promoted Gli1 SUMO modification and nucleus transfer. Mechanistically, RMRP induced GSDMD-mediated pyroptosis, proinflammatory activation, and collagen expression through the Hedgehog pathway. Notably, the mechanical stress-induced hypertrophy of LF in rabbit exhibited analogous pathological changes of LF fibrosis occurred in human and showed enhanced levels of collagen and α-SMA. Knockdown of RMRP resulted in the decreased expression of fibrosis and pyroptosis-related proteins, ultimately ameliorating fibrosis. The above data concluded that RMRP exerts a crucial role in regulating GSDMD-mediated pyroptosis of LF cells via Gli1 SUMOylation, thus indicating that targeting RMRP could serve as a potential and effective therapeutic strategy for LF hypertrophy and fibrosis.

## Introduction

Lumbar spinal canal stenosis (LSCS) is a highly prevalent disorder affecting life quality of the elderly population (1). The pathological mechanisms of LSCS result from various factors such as intervertebral disc herniation, facet joint hyperplasia, and ligamentum flavum (LF) degeneration. More importantly, degeneration of LF is considered as a significant contributing factor facilitating the progression of LSCS (2).

As is widely recognized, the LF is a significant part of the posterior column of the spine, since it covers and protects the posterior side of the dura sac (3). From a histological perspective, the normal LF is an elastic structure consisting of a large amount of elastic fibers and a small amount of collagen fibers. Nevertheless, as a result of hypertrophy, the LF displays signs of losing elastic fiber and growing collagen fibers, indicating fibrotic changes, which has been recognized as the primary pathological change of LF (4, 5). Despite this knowledge, the detailed molecular mechanisms underlying LF fibrosis are still unknown.

It has become increasingly clear that the role of inflammation has become increasingly apparent (6, 7). Similarly, LF fibrosis progresses as a consequence of chronic inflammatory response as well (8, 9). Thus, it is evident that the inflammatory response serves as a significant pathological mechanism in LF fibrosis. Pyroptosis, a newly recognized type of programmed cell death triggered by inflammasomes and orchestrated under the control of Gasdermin D (GSDMD), leads to a massive release of proinflammatory cytokines and local inflammatory response (10). Despite the strong association of pyroptosis with fibrosis in various tissues (10), there has been no reports linking pyroptosis to the pathogenesis of LF fibrosis.

In general, it is acknowledged that lncRNAs are a crucial type of RNA transcripts comprising multiple transcripts that are long over 200 nucleotides and lack the function of protein-coding (11). There is emerging evidence demonstrating that lncRNAs play vital roles in numerous biological processes and various diseases (12). Recently, it has been shown that several lncRNAs have been implicated in fibrosis diseases (13), including LF fibrosis (14). Our previous research have established that Hedgehog-Gli1 pathway is critical for driving the hypertrophy and fibrosis of LF (15). In the RIP experiment, we found RMRP physically interacted with Gli1 and was enriched in Gli1-activated LF cells. Additionally, the hypertrophic LF expressed abnormal pyroptosis-related proteins. However, the mechanism for pyroptosis activation and the downstream effects of RMRP on LF fibrosis remain poorly understood. Here, we aimed to further investigate the potential relationship and mechanism between RMRP and pyroptosis for LF fibrosis.

## Materials and methods

### Approval by the ethical community

An approval number of 2020-03-01-H02 was obtained from Nanjing Medical University’s Ethics Committee. Specimens of both human and rabbit LF were provided by the Affiliated Jiangning Hospital with Nanjing Medical University. Participation in this study was subject to written informed consent from each patient.

### Subjects

Samples were obtained from patients undergoing lumbar posterior decompressive laminectomy at the authors’ Hospital who had LSCS or lumbar disc herniation (LDH). LSCS patients were included if they met the following criteria: age 55 to 70 years, presence of spinal stenosis at L4/5 level, and MRI-measured LF thickness greater than 4mm. Samples of non-hypertrophic LF were collected from age-matched LDH patients with LF thickness below 4 mm. A number of conditions were excluded from the present study, including lumbar spondylolisthesis, spinal tumors, spinal tuberculosis, heart and kidney diseases, and skeletal dysplasia. During this study, all patients enrolled had spinal surgeries performed by three experienced spinal surgeons. A detailed information description of all enrolled patients is presented in **Table 1**.

**Table 1:** Comparison of data between two groups.

Index	LSCS group	LDH group	P value
Number of patients	18	16	
Mean age (years)	61.39±3.32	58.06±2.46	<0.05
Gender (male/female)	11/7	8/8	
Level	L4/5	L4/5	
LF thickness	4.91±0.39	2.98±0.25	<0.05
Fibrosis score	3.38±0.21	1.31±0.26	<0.05
RMRP mRNA expression	0.48±0.05	0.34±0.04	<0.05

### LF samples collection

During the surgical procedure, each patient’s LF sample used in this study was collected exclusively at the L4/5 level from the dorsal side of the LF and then used for subsequent experiments. Briefly, all LF samples were divided into three parts. One portion of the LF samples collected from surgery were rapidly frozen in liquid nitrogen to be analyzed later in a molecular biology laboratory. Histopathological analysis of another portion of the LF tissues was carried out by embedding them in paraffin following 48 hours of fixing in 4% paraformaldehyde (PFA). Each specimen was cut along the coronal plane using a paraffin microtome at the same level. As a final step, the remaining portion of tissues was immediately used for isolating primary LF cells.

### LF thickness measurement

**Figure 1:**
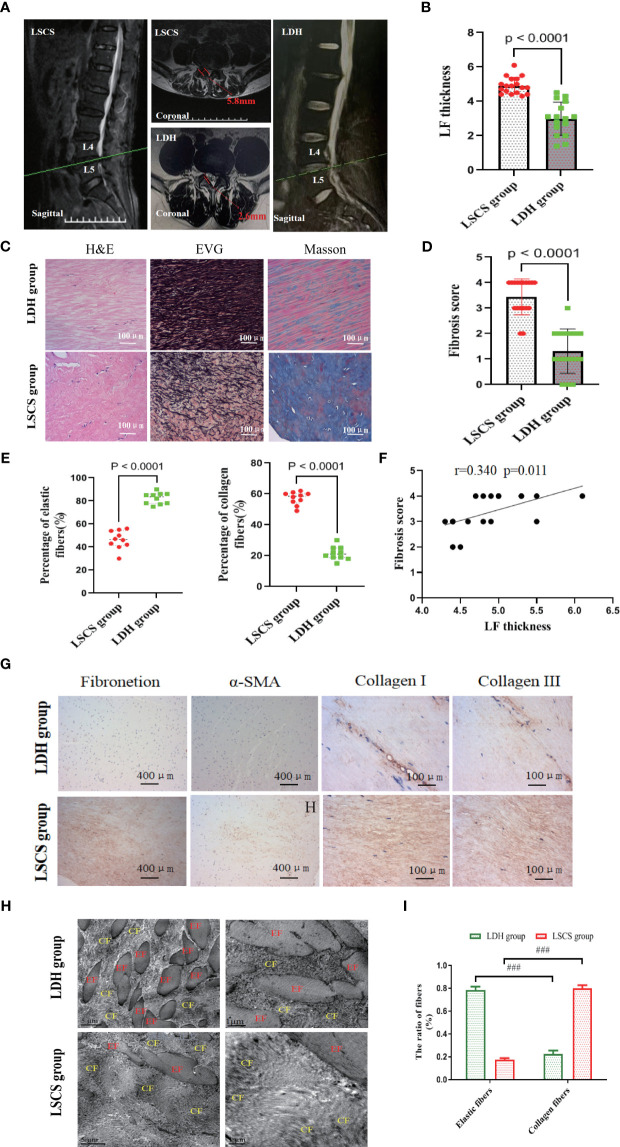
Measurement of LF thickness, and morphology and structure observation LF samples. **(A)** Representative measurement of LF thickness on the T2-weighted MRI at the facet joint level marked by the red arrow. **(B)** Comparison of LF thickness between two groups. **(C)** Images of LF samples stained with H&E, EVG, and Masson (n=8). Scale bar, 100μm. **(D)** Quantitative analysis of LF fibrosis score in two groups. **(E)** Quantitative analysis of elastic and collagen fiber ratios. (n=10). **(F)** Correlating LF thickness with fibrosis score in LSCS group. (n=18). **(G)** Images of IHC staining of fibronectin, α-SMA, collagen I, and collagen III in the LF samples of two groups. (n=6). **(H, I)** Ultrastructure observation of LF by transmission electron microscopy (n=2). Scale bar, 5μm. Mean ± SD results are presented; ^###^P < 0.001. LF, ligamentum flavum; EF, elastic fiber; CF, collagen fiber; LDH, lumbar disc herniation; LSCS, lumbar spinal canal stenosis.

All patients enrolled in this study underwent lumbar MRI scanning prior to surgery. Following the previously described method (15), T2-weighted MRI at the level of L4/5 facet joint was performed using PACS software for all patients to measure the LF thickness. The measurement of all MRI images was made by an experienced spine surgeon blind to the patients’ treatment. A three-time measurement of LF thickness for each patient was taken as the final measurement (**Figure 1A**).

### Histological and immunohistochemical staining

In order to decalcify the LF samples from humans or rabbits, they were fixed overnight in paraformaldehyde at 4°C and then transferred to 10% EDTA. Then, the specimens were cut into 4µm sections using a microtome following being embedded in paraffin. The sections were then deparaffinized, hydrated, and stained with Masson trichrome staining kits, H&E staining kits, or Verhoeff’s van Gieson (EVG) staining kits (Shanghai, China) following the instructions provided by the manufacturer. An evaluation of LF morphology and structure was conducted with H&E staining. Collagen and elastic fibers were examined using Masson staining. Masson staining and EVG staining were utilized to determine the fibrous lesion and fibrosis score of LF.

Immunohistochemical (IHC) staining was performed on the LF sections by incubating them overnight at 4°C with antibodies against fibronectin, collagen I, collagen III, ASC, Caspase-1, NLRP3, Col1a1, Col3a1, and α-SMA diluted to the manufacturers’ recommended concentrations after dewaxing and rehydrating. Afterwards, the sections were incubated with secondary antibodies (Abcam, Britain) following three washes with PBS. Finally, a laser scanning microscope (Zeiss, Germany) was utilized for visualization of the results following counterstaining with hematoxylin. Plots were generated using SPSS 17.0 software and GraphPad Prism 9.5.1.

### Transmission electron microscopy (TEM)

So as to observe the morphology and any ultrastructure changes of LF, the transmission electron microscope (Delong, America, TEM) was utilized in the present study. After being collected from the surgery, the LF specimens were immediately chopped into small pieces about 1 mm^3^ before immersing them for three hours in glutaraldehyde solution. Specimens were then washed with PBS, embedded in Epon, and sliced into about 80nm thick sections with the ultramicrotome (Leica, Germany) after being dehydrated with propylene oxide. In the end, a TEM was used to observe and analyze the sections after being stained with 2% uranyl acetate.

### LF cell isolation and culture

LF tissues from patients during the surgery were in accordance with previously described method (15). In brief, the collected LF samples were washed immediately using PBS (Gibco) following surgery, sliced into small pieces, and then digested in an incubator for one hour with 0.2% collagenase (Gibco). In the following step, the pieces were cleaned with DMEM (Gibco) and cocultured with DMEM by adding fetal bovine serum (Sigma) and penicillin/streptomycin (Sigma). The confocal microscope (XTL3230-DIC) was utilized to identify LF cells by immunofluorescence staining using antibodies from Proteintech against collagen I, collagen III, and α-SMA. In the subsequent experiment, P3 generation LF cells were collected and their supernatants were obtained for ELISAs.

### Cell viability assays

In order to determine the proliferation ability of LF cells, the MTT assay was used. Briefly, a total of 10^5^ LF cells were seeded in 96-well plates per well and cultured for 24 hours with the condition of 37°C and 5% CO^2^. Following this, an MTT working solution (Beyotime, Nanjing, China) supplemented with 80 µl of DMEM was added to every well (20 µL/well) and incubated for 4 hours at 37°C. Lastly, an absorbance measurement at 490nm was conducted by a microplate reader (CLV632-1120F0, Germany).

In this study, Annexin V-FITC Apoptosis Detection Kit (Abcam, Britain) were used for flow cytometry to analyze LF cell apoptosis. Follow the instructions, Annexin V-FITC and propidium iodide staining solutions were added and then incubated with LF cells for 20 minutes in the dark. Finally, the apoptosis of the LF cells was detected by flow cytometry using a CytoFLEX SRT device from China.

### Immunofluorescence staining

At room temperature, LF cells were first fixed for 10 minutes with 4% paraformaldehyde, followed by three wash steps with PBS. After that, cells were permeabilized for 15 minutes with 0.1% Triton, then blocked for one hour with 5% goat serum. Subsequently, incubation of the cells with antibodies against SUMOI, Gli1, collagen I, and collagen III were performed overnight at 4°C. Fluorescein isothiocyanate-conjugated (green) secondary antibodies (Abcam) against mouse IgG were then added to incubate the LF cells for one hour in the dark. DAPI (Beyotime) was used to stain the nuclei for 10 minutes. Fluorescence images were taken using an Olympus microscope.

### Adenovirus construction and Infection

The RMRP lentivirus (RMRP), negative control lentivirus (Vector), targeting DANCR lentivirus with short hairpin RNA (shDANCR), lentiviruses with control shRNA (shNC), the Gli1 lentivirus (LV-Gli1), negative control lentivirus (LV-NC) were created, packaged and tested in Guangzhou by RiboBio Co. Ltd. As described previously and directed by the manufacturer (15), the LF cells were isolated, cultured, collected and transfected. Western blot or RT-PCR were used to determine infection efficiency.

### RNA sequencing and bioinformatic analysis

Gli1 transfected LF cells and scramble control cells were collected to obtain purified total RNA. Purification of the qualified RNA was performed using the RNA Clean XP Kit (Biotopped, Beijing). The construction of Illumina sequencing libraries for lncRNA sequencing were performed according to a modified protocol for strand-specific RNA sequencing. Subsequently, A cluster was formed by loading the libraries onto cBot (Illumina, USA) and sequenced on the HiSeq X 10 platform at the Nanjing medical Corporation. Hisat2 (Version 2.1.0) was used to aligned RNA-seq clean reads to human reference genome sequence assembly (Homo sapiens. GRCh38). After mapping, differentially expressed genes were identified using edge R software with the screening criteria of a threshold of P < 0.05 and fold change ≥1.5. Finally, the differentially expression genes were subjected to GO analysis using DAVID software.

### RNA pull-down assay

RNA pull-down assays were conducted as directed by the manufacturer using the RNA Pull-Down Kit (Termo Scientific). RMRP binding complexes were captured by lysing LF cells with precooled capture buffer, followed by incubation with biotin-labeled RMRP. Further analysis was then conducted by extracting the total RNA bound to the complexes.

### RT-PCR

TRIzol reagent (Invitrogen,USA) was utilized for the isolation of total RNAs from rabbit and human LF samples, and LF cells. The total RNAs were reverse-transcribed into cDNA by using a Takara Kit according to the manufacturers’ protocol. After that, gene expression levels were measured with a Real-Time System for Thermal Cycler Dice (Takara). Using 2^-ΔΔCt^ method, the relative levels of mRNA expression were calculated by GAPDH as an internal control. In **Table 2**, the primers with specific designs and their sequences are listed.

**Table 2:** RT-PCR primers used in this study.

Primer	Sequence
Gli1	Forward: 5′-TTCCTACCAGAGTCCCAAGT-3′ Reverse: 5′ -CCCTATGTGAAGCCCTATTT-3′
RMRP	Forward: 5′-ACTCCAAAGTCCGCCAAGA-3′ Reverse: 5′ -TGCGTAACTAGAGGGAGCTGAC-3′
GSDMD	Forward: 5'-TGAATGTGTACTCGCTGAGTGTGG-3' Reverse: 5'-CAGCTGCTGCAGGACTTTGTG-3'
α-SMA	Forward: 5′ -CATTTGGTCAGAAGACGGTTG-3′ Reverse: 5′ -GACCTGGAGTTCTCACTTTCATC-3′
Caspase-1	Forward: 5′ -ACAGGCATGACAATGCTGCT-3′ Reverse: 5′ -GCTGTC-AGAGGTCTTGTGCT-3′
ASC	Forward: 5′ -TGACGGATG-AGCAGTACCA-3′ Reverse: 5′ -GGCCTGGAGGAGCAAGT-3′
NLRP3	Forward: 5′ -GATCTTCGCTGCGATCAACA-3′ Reverse: 5′ -GGGATTCGAAACACGTGCATTA-3′
IL-1β	Forward: 5′ -CCAGGGACAGGATATGGAGCA-3′ Reverse: 5′ -TTCAACACGCAGGACAGGTACAG-3′
IL-18	Forward: 5′ -CTGCCACCTGCTGCAGTCTA-3′ Reverse: 5′ -TCTACTGGTTCAGCAGCCATCTTTA-3′
Collagen I	Forward: 5′ -ATGCCTGGTGAACGTGGT-3′ Reverse: 5′ -AGGAGAGCCATCAGCACCT-3′
Collagen III	Forward: 5′ -CGCTCTGCTTCATCCCACTATTA-3′ Reverse: 5′ -ATTTGGCATGGTTCTGGCTTC-3′
GAPDH	Forward: 5′ -AGAAGGTGGTGAAGCAGGCGTC-3′ Reverse: 5′ -AAAGGTGGAGGAGTGGGTGTCG-3′
Elastin	Forward: 5′ -GCCTGGGCTTGGAGTTGGTGC-3′ Reverse: 5′ -CACGCCTCCCGCTCCGTATTTC-3′
Col1a1	Forward: 5′ -CAAGACCACCAAGACCTCCCG-3′ Reverse: 5′ -GTCTGGGTTGTTTGTCGTCTGTTTC-3′
Col3a1	Forward: 5′ -CGAGCCTCCCAGAACATCAC-3′ Reverse: 5′ -GAGCAGCCATCCTCCAGAAC-3′

### Western blotting

The Protein Extraction Sample Kit (Sigma) was used to isolate proteins from human LF samples, rabbit LF samples, and LF cells as previous described (15). After separation in 10% SDS-PAGE gels, the samples were electroblotted onto the PVDF membrane (Sigma),. The membranes were blocked in Blocking Buffer (Sigma) and then incubated at 4°C overnight with primary antibodies against SUMO1 (1:1000, ab32058, Abcam), ASC (1:1000, ab283684, Abcam), Gli1 (1:1000, ab167388, Abcam), Caspase-1 (1:500, ab238972, Abcam), Cleaved Caspase-1 (1:1000, bs-1247R, Bioss), GSDMD-N (1:500, ab215203, Abcam); GSDMD (1:1000, ab219800, Abcam), collagen I (1:1000, 14695-1-AP, Proteintech), collagen III (1:1000, bs-0549R, Bioss), NLRP3 (1:500, ab263899, Abcam), GAPDH (1:1000, AP0063, Bioworld), and α-SMA (1:1000, 55135-1-AP, Proteintech). Then, western blotting kit (Bioworld) was used to detect signals of each blotting band after incubation with secondary antibodies (1:1000, SA00001-2, Proteintech).

### Enzyme-Linked Immunosorbent Assays

Supernatants from LF cells were collected and used to detect the local concentrations of IL-1β and IL-18. Enzyme-linked immunosorbent assays (ELISAs) kits (E-EL-H0253c, Elabscience, China) were employed following the manufacturer’s protocol to carry out the measurements.

### Rabbit LF degeneration and hypertrophy model

Animal Ethics Committee of Nanjing Medical University approved all the animal experiments. An animal experiment center at Nanjing Medical University provided fifteen male New Zealand rabbits, each weighing about 2.5-3.0 kilograms. The hypertrophic LF rabbit model was established as previously described (15). Randomly, the rabbits were assigned to three groups: the control group with no treatment (n=5), the LF hypertrophy (LFH) group (n=5) + shNC group, and the LFH + AAV-shRMRP group (n=5). After eight weeks of modeling, a midline incision about 6cm was made under anesthesia. Subsequently, the lumbosacral fascia was cut from one side of the mammillary process and the multifidus was detached to expose the LF at L3/4 level. Additionally, AAV-shNC or AAV-shRMRP (4µl, 1×10^12^vg/ml) from Jima Pharmaceutical Technology (Shanghai, China) was injected into the LF using microliter micro-syringes under an operating microscope (Hamilton, Switzerland). After the rabbits had been modeled for 8 weeks, they were sacrificed, and LF samples from the L4/5 level were obtained for the subsequent experiments. A free diet and water were provided to all rabbits enrolled in the study.

### Statistical analysis

Unless otherwise noted, all data are presented in the form of mean ± SD. Student’s t-tests and one-way ANOVAs were employed to analyze differences between groups. For analyzing the correlation between two indices, Pearson correlation coefficient was used. Statistical significance was considered by P < 0.05. SPSS 22.0 software (IBM, Chicago, IL) was used to perform statistical analysis.

## Results

### LF thickness measurement, fibrosis score and structural analysis

As illustrated in **Table 1**, The current study included 18 LSCS patients and 16 LDH patients. In terms of age, sex, or LF level, statistically insignificant were found between the two groups (P>0.05).

At the level of L4/5 facet joint, representative coronal T2-weighted MRI images were used to measure LF thickness (**Figure 1A**). Previous research has indicated that LF thickness is typically greater than 4 mm in patients with LSCS (15, 16). Based on the representative MRI scans shown in **Figure 1A**, LF thickness higher than 4 mm usually results in compression of the dural sac and nerve root, ultimately leading to spinal stenosis. Nevertheless, the LF from LDH did not oppress the dural sac, which was usually less than 4 mm thick. Consistent with previous studies, the LF thickness of LSCS group was 4.91 ± 0.39 mm, compared to 2.98 ± 0.25 mm in control group (**Figure 1B** and **Table 1**). Evidence from previous studies has indicated structural abnormalities in LF from LSCS patients (2, 5). As revealed by EVG and H&E staining in this study, LF in LSCS group had few elastic fibers and abundant collagen fibers. Morphologically, elastic fibers in LSCS group showed unevenness, fragmentation, irregular arrangement, and partial absence. In contrast, a considerable amount of elastic fibers were organized regularly in LDH group, whereas a small portion of collagen fibers were present (**Figure 1C**).

In addition, the LF fibrosis score was 3.38 ± 0.21 in LSCS group, whereas it was 1.31 ± 0.26 in LDH group (**Figure 1D**, P< 0.05) based on Masson staining analysis. The characteristics of fibrosis were also evidenced by quantitative analysis showing a reduction in the volume ratio of elastic fibers and a greater volume ratio of collagen fibers compared to normal LF based on Masson staining and EVG staining (**Figure 1E**). Furthermore, Positive correlations were found between the fibrosis score and LF thickness (P<0.05) (**Figure 1F**), suggesting that LF hypertrophy is characterized by fibrosis. Additionally, it was observed that the LSCS group accumulated more fibrosis-associated proteins such as fibronectin, α-SMA, collagen I, and collagen III than the LDH group as detected by IHC (**Figure 1G**).

Finally, TEM was utilized to observe the ultrastructure of LF. In brief, on the coronal plane, elastic fibers in the LSCS group exhibited predominantly long shuttle or irregular shapes. Conversely, there was a large presence of elliptical or short shuttle shapes in the LDH group (**Figure 1H**). The LDH group displayed massive regularly arranged elastic fibers and minimal collagen fibers. As opposed to LDH group, LSCS group showed significant degradation of elastic fibers content, whereas it had abundant collagen fibers (**Figure 1I**).

### RMRP was elevated in hypertrophic LF and linked to LF hypertrophy

**Figure 2:**
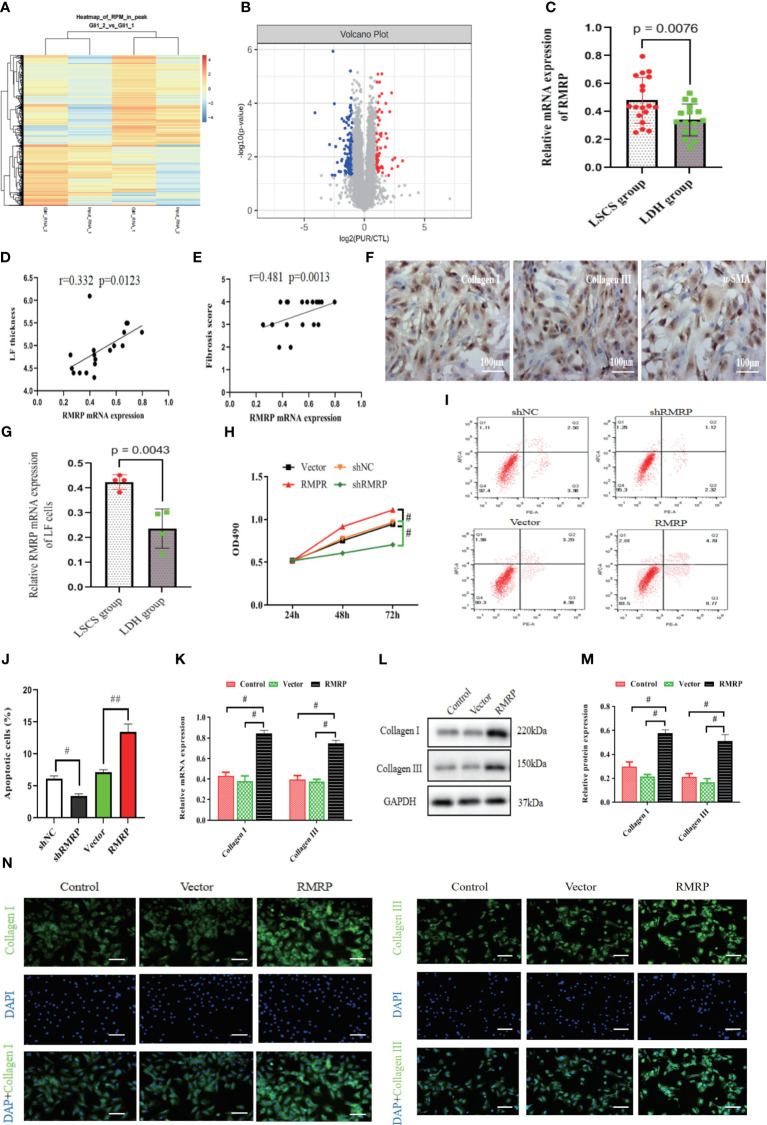
RMRP was elevated in hypertrophic LF and linked to LF hypertrophy and fibrosis. **(A)** Heatmap displaying genes with at least a 2-fold change in Gli1-transfected or control LF cells. **(B)** The volcano plots showed lncRNA expression in Gli1-transfected or control LF cells (n=3). Red dots in the volcano plots represent upregulated RNAs, while blue represent downregulated RNAs. **(C)** RT-PCR detecting mRNA levels of RMRP in LF tissues (LSCS group: n=18; LDH group: n=16). **(D, E)** Analyzing the correlation between RMRP level and LF thickness **(D)** and fibrosis score **(E)**. **(F)** Identifying the phenotype of LF cells; scale bar, 100μm. **(G)** Quantitative analysis of the RMRP level by RT-PCR in the LF cells of two groups. **(H)** Impact of RMRP on LF cell growth measured with MTT (n=3). **(I, J)** Effect of RMRP on LF cell apoptosis detected by flow cytometry (n=3). **(K–N)** RMRP’s effect on collagen expression in LF cells detected by RT-PCR **(K)**, western blot **(L, M)** and immunofluorescence staining (N, n=3); Scale bar, 100μm. ^#^P < 0.05, ^##^P < 0.01. LDH, lumbar disc herniation; LSCS, lumbar spinal canal stenosis; LF, ligamentum flavum.

In order to explore the potential molecular mechanism underlying LF fibrosis, our study specifically examined Hedgehog-Gli1 signaling pathway, which has previously been implicated in LF fibrosis (15) and fibrosis of other organs (17). In line with previous findings, we conducted a RIP experiment to analyze the expression profiles of lncRNA in LF cells transfected with Gli1 or scramble control using sequencing analysis of RNA binding to Gli1. Specifically, RNA-seq was performed in Gli1 transfected LF cells (n=3) and control LF cells (n=3), and differential expression of RNAs was determined using |logFC|>1.0 and P <0.05 as a screening criterion. The findings of our study indicate that there were 1009 long non-coding RNAs (lncRNAs) that exhibited differential expression levels between the two groups, with 608 upregulated and 401 downregulated (**Figures 2A, B**). Among the upregulated genes, LncRNA RMRP exhibited a threefold increase. Furthermore, we observed a significant upregulation of lncRNA RMRP in LF specimens of LSCS group as compared to the group with LDH, as confirmed by quantitative RT-PCR (**Figure 2C**). Pearson correlation coefficient analysis revealed that there existed a strong positive correlation between RMRP levels and the thickness of LF as well as fibrosis score in hypertrophic LF of LSCS group (**Figures 2D, E**). Furthermore, microscopic observation and immunofluorescence identified cells obtained from the LF sample with typical fibroblast morphology and marked expression of cell markers of collagen I, collagen III, and α-SMA, indicating high purity of LF cells (**Figure 2F**). Subsequently, expression levels of the RMRP were detected in LF cells from LDH and LSCS using RT-PCR. Consistent with the results obtained from LF tissues, the RMRP level was markedly increased in LF cells of LSCS group compared to LDH group (**Figure 2G**). Collectively, these data indicated that abnormal RMRP expression occurred in hypertrophic LF tissues.

To validate the impact of RMRP on the viability of LF cell, an analysis was performed to evaluate its effect on growth and survival of LF cells. In **Figure 2H**, there was a significant increase in cell proliferation in RMRP over-expressed LF cells compared to the control. Moreover, flow cytometry assay indicated a notably lower percentage of apoptosis in LF cells over-expressing RMRP than in the control cells (**Figures 2I, J**). To further explore the influence of RMRP on fibrogenesis in LF cells, we analyzed its effect on the levels of fibrosis-associated proteins. As illustrated in **Figures 2K–M**, up-regulation of RMRP clearly elevated both mRNA (**Figure 2K**) and protein (**Figures 2L, M**) levels of collagen types I and III in LF cells. This finding was further supported by immunofluorescence results (**Figure 2N**). In summary, our findings suggested a strong correlation between RMRP and the pathogenesis of LF hypertrophy.

### RMRP stimulated fibrogenesis *in vitro* via the activation of Hedgehog-Gli1 signaling

**Figure 3:**
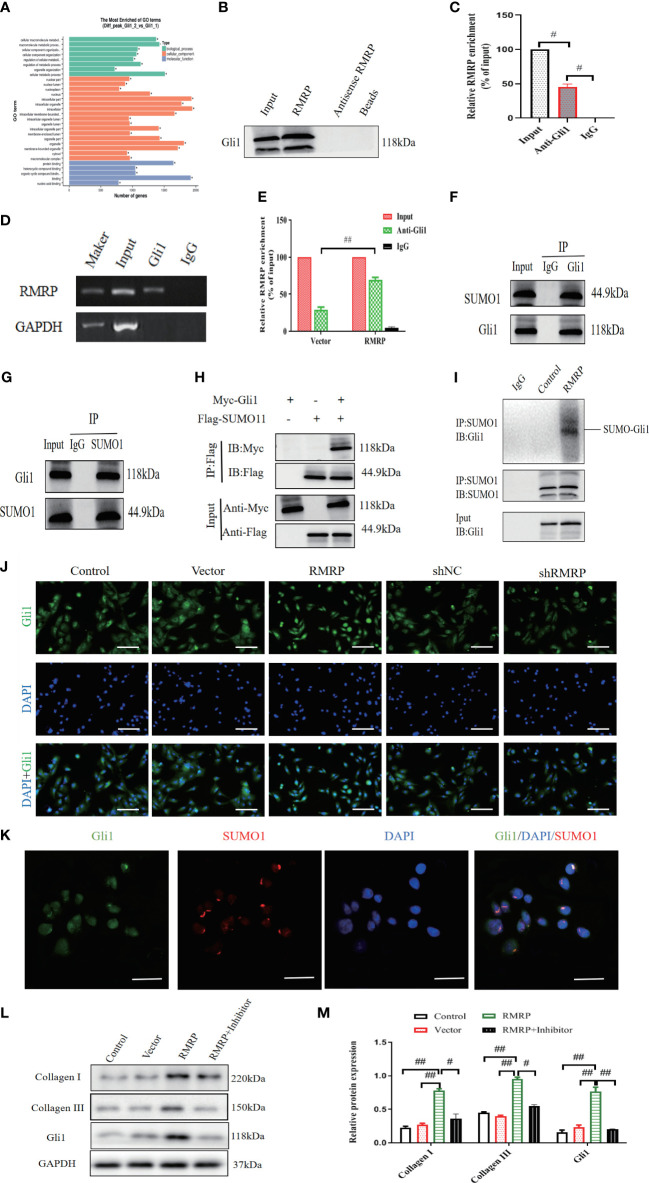
RMRP stimulate fibrogenesis *in vitro* through the activation of Hedgehog-Gli1 signaling. **(A)** GO analysis for dysregulated genes in LF cells. **(B)** Western blot analysis of Gli1 and RMRP association in RNA pull-down assay. **(C)** RIP assay using Gli1 antibody, detection of RMRP or GAPDH by specific primers, and quantification of RIP enrichment **(D, E)** Increased RMRP expression enhanced its interaction with Gli1, as shown by RIP assay with Gli1 antibody. (n=3). **(F, G)** Co-IP **(F)** and reverse Co-IP **(G)** were used to assess interaction of Gli1 with SUMO1 examined by immunoblotting with antibodies (n=3). **(H)** HEK 293T cell lysate transfected with Myc-Gli1 and Flag-SUMO1 was immunoprecipitated with anti-Flag and immunoblotted with anti-Flag and anti-Myc (n=3). **(I)** Lysates from LF cells transfected with LV-control or LV-RMRP were immunoprecipitated and analyzed with specific antibodies to study the impact of RMRP on Gli1 SUMOylation (n=3). **(J)** RMRP’s influence on Gli1 translocation to the nucleus in LF cells, measured with immunofluorescence. Scale bar, 100μm. **(K)** Examining the co-localization of Gli1 and SUMO1 proteins using immunofluorescence analysis. Scale bar, 20μm. **(L, M)** Western blot analysis of the impact of Hedgehog pathway on RMRP-induced collagen expression. ^#^P < 0.05, ^##^P < 0.01. LF, ligamentum flavum.

Next, we investigated the possible mechanism through which activated RMRP facilitate fibrogenesis in an *in vitro* setting. As summarized in **Figure 3A**, GO pathway analysis was conducted on dysregulated genes in Gli1-transfected or control LF cells. The results revealed that the pathways involving protein binding and cellular component were notably significant for the dysregulated genes in the Gli1-over-expressed LF cells. Additionally, prior research has suggested that lncRNAs modulate their biological activity through interactions with particular proteins (18, 19). In order to elucidate the regulatory mechanism of RMRP with Gli1, a RNA pull-down assay was performed to identify proteins interacting with RMRP. The analysis indicated that Gli1 was the most prominently enriched RMRP-binding protein. In brief, western blot result confirmed the presence of abundant Gli1 in RMRP-pull-down lysates (**Figure 3B**).

Furthermore, the interaction between RMRP and Gli1 was confirmed through RNA immunoprecipitation (RIP) utilizing antibodies specific to Gli1 in LF cells. Our results demonstrated a significant enrichment of RMRP RNA when utilizing the Gli1 antibodies (**Figure 3C**). Additionally, over-expression of RMRP markedly enhanced the interaction between RMRP and Gli1 as detected by RIP-PCR (**Figures 3D, E**). Altogether, our findings established a direct interaction between RMRP and Gli1.

To study the mechanism by which RMRP triggers Hedgehog-Gli1 during fibrogenesis, we aimed to determine whether RMRP might regulate the SUMOylation of Gli1. Initially, we used Co-IP to examine the protein interactions with Gli1. As illustrated in **Figure 3F**, Co-IP analysis revealed that Gli1 precipitated SUMO1 in LF cells. Subsequent reverse Co-IP also confirmed a significant precipitation of Gli1 by SUMO1 in LF cells (**Figure 3G**). Additionally, we conducted a Co-IP using epitope-tagged proteins and observed efficient co-precipitation of Flag-labeled SUMO1 and Myc-labeled Gli1 in 293T cells (**Figure 3H**). Next, we found that the over-expression of RMRP effectively increased Gli1 SUMOylation in LV-RMRP transfected LF cells as compared with the control (LV-NC, **Figure 3I**). These data demonstrated the interaction between Gli1 and SUMO1. Furthermore, immunofluorescence staining was utilized to investigate the effect of RMRP on Gli1 expression and nuclear translocation. Results revealed that in control group, Gli1 was mainly found in the cytoplasm, not the nucleus. However, in RMRP-transfected LF cells, a majority of Gli1 translocated to the nucleus (**Figure 3J**), suggesting that RMRP activated the Hedgehog-Gli1 pathway by inducing the Gli1 nuclear transfer. Immunofluorescence co-localization analysis suggested that Gli1 and SUMO1 combined and co-localized in the nucleus (**Figure 3K**), indicating that RMRP facilitated nuclear translocation of Gli1 through regulating its SUMO modification.

Next, we aimed to investigate how RMRP induced collagen expression. In light of the above data, we theorized that RMRP could upregulate collagen expression through the Hedgehog signaling. To validate the hypothesis, We studied how RMRP affects collagen expression by using a Hedgehog inhibitor (cyclopamine) on LF cells. The results of our study indicate that the elevated collagen expression mediated by RMRP was markedly reduced by treatment with cyclopamine (**Figures 3M, N**), as detected by western blot analysis. Collectively, our findings indicate that RMRP contributes to the pathological advancement of fibrogenesis *in vitro* through the induction of Gli1 SUMOylation.

### RMRP promoted pyroptosis *in vitro* by activating Hedgehog-Gli1 signaling

**Figure 4:**
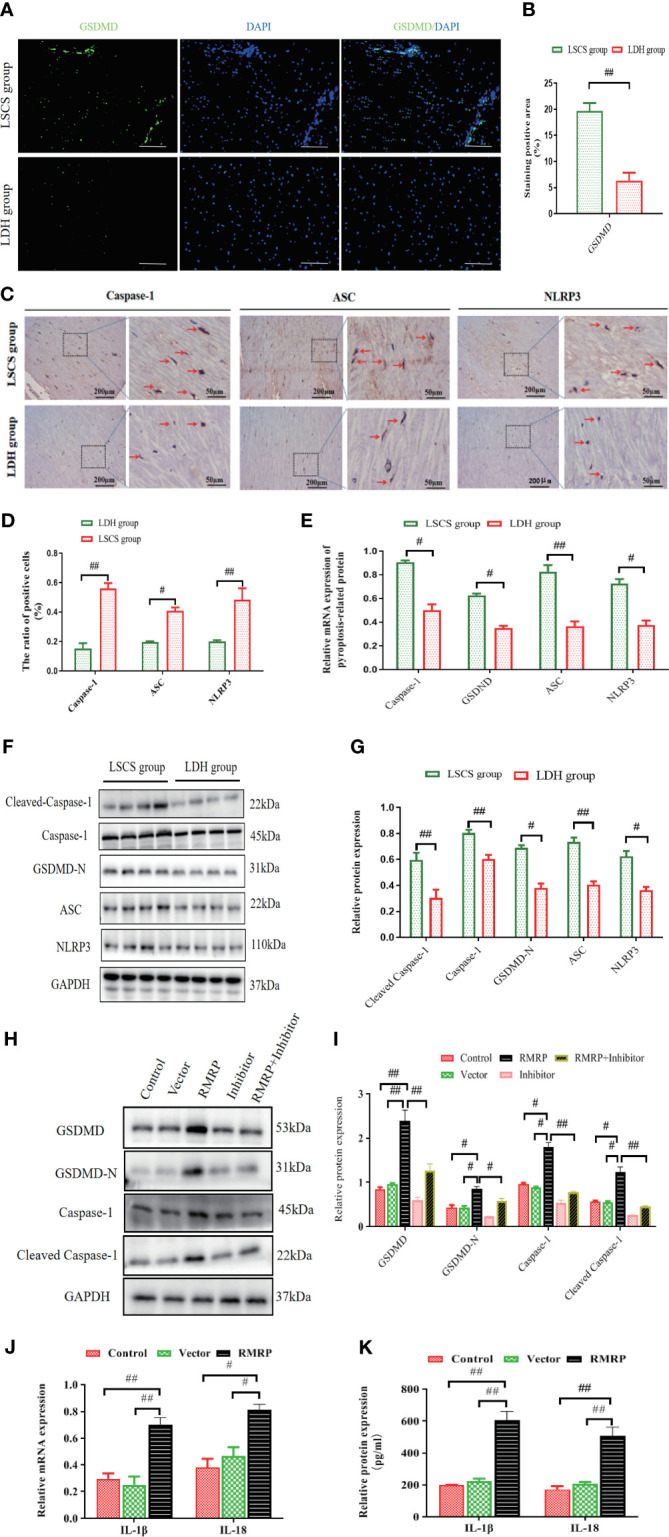
RMRP facilitated pyroptosis *in vitro* by activating Hedgehog-Gli1 pathway. **(A)** GSDMD in human LF samples detected by immunofluorescence. Scale bar, 200 µm. **(B)** Quantitative analysis of GSDMD expression area. **(C)** Images showing IHC staining of Caspase-1, ASC, and NLRP3 in LF samples. **(D)** Results of quantitative analysis of the Caspase-1-, ASC-, and NLRP3-positive areas. **(E)** RT-PCR results of Caspase-1, GSDMD, ASC, and NLRP3 in LF samples (n=8). **(F, G)** Western blot results of pyroptosis-related protein levels (C-Caspase-1, Caspase-1, ASC, GSDMD-N, and NLRP3; n=8). **(H, I)** RMRP’s impact on the expression of pyroptosis-related proteins in LF cells with or without cyclopamine. (5μM). **(J, K)** The effect of RMRP on the mRNA **(J)** and protein **(K)** expressions of inflammatory mediators (IL-1βand IL-18) in LF cells. Results are presented as means ± standard deviations; ^#^P<0.05, ^##^P<0.01. LSCS, lumbar spinal canal stenosis; LDH, lumbar disc herniation.

As is known, pyroptosis is presently characterized as a form of programmed necrosis mediated by gasdermin (20). The protein Gasdermin D (GSDMD), which is cleaved by caspase1/4/5/11, plays a crucial role in the induction of pyroptosis following inflammasome activation. Emerging evidence indicates that pyroptosis is a significant contributor to the pathogenesis of fibrosis in multiple organ systems (10, 21, 22). As such, we noted a marked up-regulation of GSDMD fluorescence intensity in LF specimens of LSCS group compared to LDH group (**Figures 4A, B**). Furthermore, an IHC analysis was performed on LF tissues in order to evaluate the cellular origins of Caspase-1, ASC, and NLRP3. The findings indicated a statistically significant increase in the number of positive cells in LSCS group compared to LDH group (**Figures 4C, D**). In accordance with IHC results, elevated levels of mRNA (**Figure 4E**) and protein expression (**Figures 4F, G**) of Caspase-1, C-Caspase-1, NLRP3, ASC, and GSDMD-N were also detected in LSCS group compared to LDH group. In conclusion, as a result of these data, it appears that pyroptosis may contribute to LF fibrosis pathogenesis.

To further explore the functional significance of the interaction between RMRP and pyroptosis, LF cells were transfected with lentivirus overexpressing RMRP or a scramble control, with or without the Hedgehog signaling inhibitor (cyclopamine) (15). It was found that RMRP overexpressing promoted protein levels of C-Caspase-1, Caspase-1, GSDMD-N, and GSDMD in the LF cells. These effects were markedly attenuated by cyclopamine as determined by western blot analysis. Moreover, the levels of both IL-18 and IL-1β in the supernatant, as well as the mRNA levels in LF cells transfected with RMRP overexpressing lentivirus were also markedly elevated. Collectively, our results suggested that RMRP induced LF cell pyroptosis through its interaction with Hedgehog signaling.

### RMRP knockdown alleviated LF hypertrophy and fibrosis caused by mechanical stress *in vivo*

**Figure 5:**
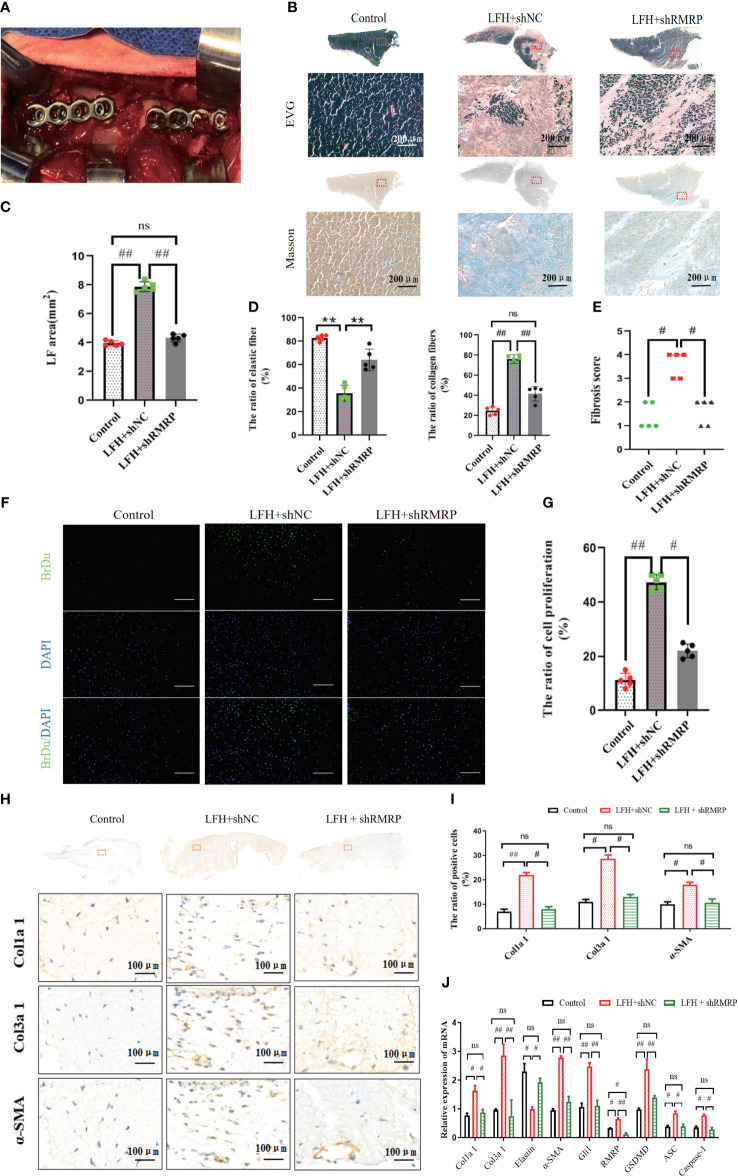
RMRP knockdown alleviated LF hypertrophy and fibrosis induced by mechanical stress *in vivo*. **(A)** Rabbit with fused spine, lateral view. **(B)** Images of LF stained with EVG and Masson. Scale bar, 200μm. **(C)** Quantitative analysis of the LF areas based on Masson staining at the same level. **(D)** Analysis of the area ratios of elastic and collagen fibers based on EVG staining. **(E)** Comparison of the fibrosis scores in the three groups based on the Masson staining. **(F, G)** The number of proliferating cells in three groups (Brdu, green; DAPI, blue). Scale bar, 200 µm. **(H)** Images of IHC staining of Col1a1, Col3a1, and a-SMA in LF specimens. **(I)** Quantitative analysis of Col1a1, Col3a1, and a-SMA-positive cells. **(J)** RT-PCR detection of the fibrosis and pyroptosis related genes (Col1a1, Col3a1, Elastin, a-SMA, Gli1, RMRP, GSDMD-N, ASC, and Caspase-1; n=5). ^#^P<0.05, ^**^P < 0.01, ^##^P<0.01. LFH, ligamentum flavum hypertrophy; ns, no statistical significance.

Furthermore, to validate the impact of RMRP on LF hypertrophy and fibrosis *in vivo*, we constructed a rabbit LF hypertrophy model by fixing adjacent L2-3 and L4-5 segments to concentrate stress on L3-4 level as previously described (15) (**Figure 5A**). The RNAi virus targeting RMRP (AAV-shRMRP) and its corresponding control (AAV-shNC) were synthesized and subsequently administered into L3-4 level of LF via a microinjection technique. All rabbits were assigned to one of three groups through random selection: the control group received no treatment, the LF hypertrophy (LFH) model group + shNC, and the LFH + AAV-shRMRP group. The findings from the RT-qPCR analysis confirmed a significant decrease in RMRP expression in LF samples from LFH + shRMRP group when compared to LF tissues treated with LFH + shNC group or control group (**Figure 5J**).

As anticipated, the rabbit model of LF hypertrophy demonstrates a high degree of concordance with the clinical and pathological characteristics observed in humans. Briefly, our findings indicated that the LF at L3-4 level in LFH + shNC group exhibited greater thickness compared to control group, and this effect was mitigated by AVV-shRMRP intervention in LFH + shRMRP group (**Figures 5B, C**). Consistent with prior studies (15, 23), our analysis of Masson staining and EVG staining indicated that the hypertrophic LF morphology and structure in rabbits closely resembled that observed in humans (**Figure 5B**). These findings indicated that the lumbar LF underwent degeneration and hypertrophy when subjected to prolonged mechanical stress. In addition, there was a notable reduction in elastic fibers and an elevation of collagen fibers in the LF samples of LFH + shNC group compared to control group; however, this fibrosis effect was weakened by AVV-shRMRP treatment (**Figure 5D**). Similarly, the fibrosis score exhibited a similar trend (**Figure 5E**).

In order to clarify the impact of RMRP on LF cell function, we analyzed cellular alterations in rabbit LF specimens. As illustrated in **Figures 5F, G**, a notable rise in the quantity of BrdU-positive cells was observed in LFH + shNC group in comparison to control group; however, this increase was attenuated by AVV-shRMRP intervention in LFH + shRMRP group. Furthermore, IHC staining revealed a marked upregulation of the fibrosis-related genes (Col1a2, Col3a1, and α-SMA) at L3-4 level of LF in LFH + shNC group compared to control group; however, these levels were markedly reduced in LFH + shRMRP group compared to LFH + shNC group (p<0.01) (**Figures 5H, I**). Additionally, RT-PCR analysis of fibrosis-related genes and pyroptosis-related genes showed that mechanical stress notably increased the expressions of Gli1, Col1a2, Col3a1, α-SMA, GSDMD, ASC, and Caspase-1 in LF hypertrophy tissues in LFH + shNC group; whereas there was a notable decrease in the expression of elastin mRNA. These effects were markedly mitigated by AVV-shRMRP treatment (**Figure 5J**) in LFH + shRMRP group (**Figure 5J**). The data collectively indicated that RMRP played a pro-fibrotic role in LF hypertrophy, suggesting that targeted suppression of RMRP expression may serve as a promising therapeutic approach for addressing LF hypertrophy and fibrosis.

## Discussion

In the present investigation, we provide novel evidence demonstrating that RMRP initiates hypertrophy and fibrosis of the LF by activating the Hedgehog-Gli1 signaling and promoting ECM accumulation through the induction of pyroptosis in LF cells. Furthermore, our research indicates that the suppression of RMRP can effectively reduce LF fibrosis caused by mechanical stress in the rabbit model, suggesting a promising therapeutic approach for mitigating hypertrophy and fibrosis in LSCS patients.

As widely recognized, LSCS predominantly occurs in the elderly demographic and exerts a substantial impact on human health and longevity (1, 24). LF hypertrophy has been has been identified as a significant factor in the pathogenesis and progression of LSCS (9, 15, 25). Our study found that the LF was obviously thicker in LSCS group compared to LDH group. Additionally, consistent with numerous previous studies (25–27), hypertrophic LF showed fibrotic alterations typified by accumulation of collagen fiber and loss of elastic fiber. Furthermore, Pearson correlation coefficient analysis demonstrated a positive correlation between fibrosis score and LF thickness in LSCS patients, indicating that LF fibrosis was the primary pathology responsible for LF hypertrophy. Yet, the specific molecular mechanism underlying this process has not been fully elucidated.

Our previous findings have demonstrated that Hedgehog-Gli1 pathway is a vital driver of LF hypertrophy and fibrosis (15). But the precise mechanism by which Hedgehog-Gli1 is activated remains poorly understood. The swift advancement of high-throughput sequencing technology in bioinformatics analysis has rendered RNA-seq a potent tool for elucidating the pathogenesis of diseases. Accumulating evidence has shown that a variety of lncRNAs have been identified in many tissues and participated important roles in various biological processes (28, 29). Recently, numerous studies have demonstrated that certain lncRNAs are implicated in various fibrotic diseases (30–32). Nevertheless, there is limited understanding regarding the role and mechanism of lncRNAs in LF hypertrophy. In this study, utilizing mRNA sequencing and analysis, we identified a physical interaction between lnc-RMRP and Gli1, with RMRP showing enrichment in Gli1-activated LF cells. Moreover, quantitative RT-PCR analysis revealed a significant up-regulation of RMRP in LF tissues of LSCS group. *In vitro* experiments further revealed that RMRP promoted LF cell proliferation, inhibited apoptosis and aggravated collagen accumulation, indicating its association with LF hypertrophy and fibrosis.

Subsequently, Our study delved into the potential involvement of RMRP in the LF fibrosis and its underlying molecular mechanisms. Previous research has indicated that RMRP, a long non-coding RNA expressed in various human and murine tissues, encodes the RNA component of mitochondrial RNA processing endoribo-nuclease (33) and exerts a significant role in the onset of fibrosis in various organs and tissues (34, 35). As it is reported, the Hedgehog signaling pathway plays a crucial role in tissue remodeling in various fibrotic diseases, functioning under both physiological and pathological conditions (15, 36, 37). As one of the downstream transcription factors within the Hedgehog signaling, activated Gli1 serves as a significant indicator of this pathway activity. Upon activation, Gli1 translocates to the nucleus, leading to the transcription of downstream Hedgehog genes (15, 38–40). In a manner consistent with our prior research, the present study revealed the presence of Hedgehog signaling in LF fibrosis. Utilizing an RNA pull-down assay, we successfully identified Gli1 as the predominant RMRP-binding protein. Furthermore, we found that RMRP facilitated the nuclear translocation of Gli1 and exacerbated collagen accumulation through the Hedgehog signaling pathway, suggesting that Hedgehog-Gli1 signaling axis is a target of RMRP and mediates its fibrotic effects.

It is evident that SUMOylation modification is a form of ubiquitinated protein post-translational modification process that responds to environmental influences and is closely associated with the initiation and progression of diverse diseases including cancer and many degenerative diseases (41). As a significant method of protein modification, SUMOylation can regulate the activity, function, and localization of various modified proteins. Recent studies provide increasing support for SUMOylation as a viable therapeutic target treating fibrotic diseases through the regulation of transcription factors and key mediators (41–43). In this study, through Co-IP analysis, we observed that Gli1 bound with SUMO1 and co-localized in the nucleus. Furthermore, we found RMRP effectively increased Gli1 SUMOylation in LV-RMRP transfected LF cells. In short, we concluded that RMRP activated Hedgehog pathway through the SUMOylation of Gli1.

Pyroptosis, a form of programmed cell death characterized by the stimulation of inflammasomes and the release of pro-inflammatory factors, is a recently identified phenomenon associated with significant biochemical alterations. Gasdermin D (GSDMD) plays a vital role in inducing pyroptosis and amplifying the inflammatory response (20–22). Interestingly, a growing body of evidence indicates that inflammation is increasingly recognized as a key contributor to fibrosis pathogenesis (44–46). More recently, increasing studies have demonstrated important roles of pyroptosis in fibrotic diseases (10, 47). Nevertheless, the precise contribution of pyroptosis to LF fibrosis remains unclear. Here, we revealed that the levels of pyroptosis-related proteins were increased in hypertrophic LF tissues, indicating the association of pyroptosis with LF hypertrophy. Furthermore, functional experiments showed that RMRP promoted the expressions of pyroptosis-related proteins via Hedgehog signaling. Additionally, the rabbit model further confirmed the important role of RMRP/Hedgehog-Gli1/pyroptosis profibrotic axis in LF hypertrophy.

Several limitations exist in our study. Firstly, RMPR expressions were only detected in 18 human LF samples from the group with LSCS and 16 from the LDH group, indicating a need for a larger size to validate our findings in future experiments. Secondly, as previously described, the fibrosis process is intricate and encompasses numerous distinct molecular and cellular mechanisms that contribute to the progression. Whether other lncRNAs play roles in LF hypertrophy remains unknown. Thirdly, we demonstrated that RMPR could ameliorate mechanical stress-induced LF hypertrophy by injecting AAV-shRMRP into LF using a microinjector *in vivo*; however, the most effective concentration of AAV-shRMRP remains unclear and this warrants further investigation.

**Figure 6:**
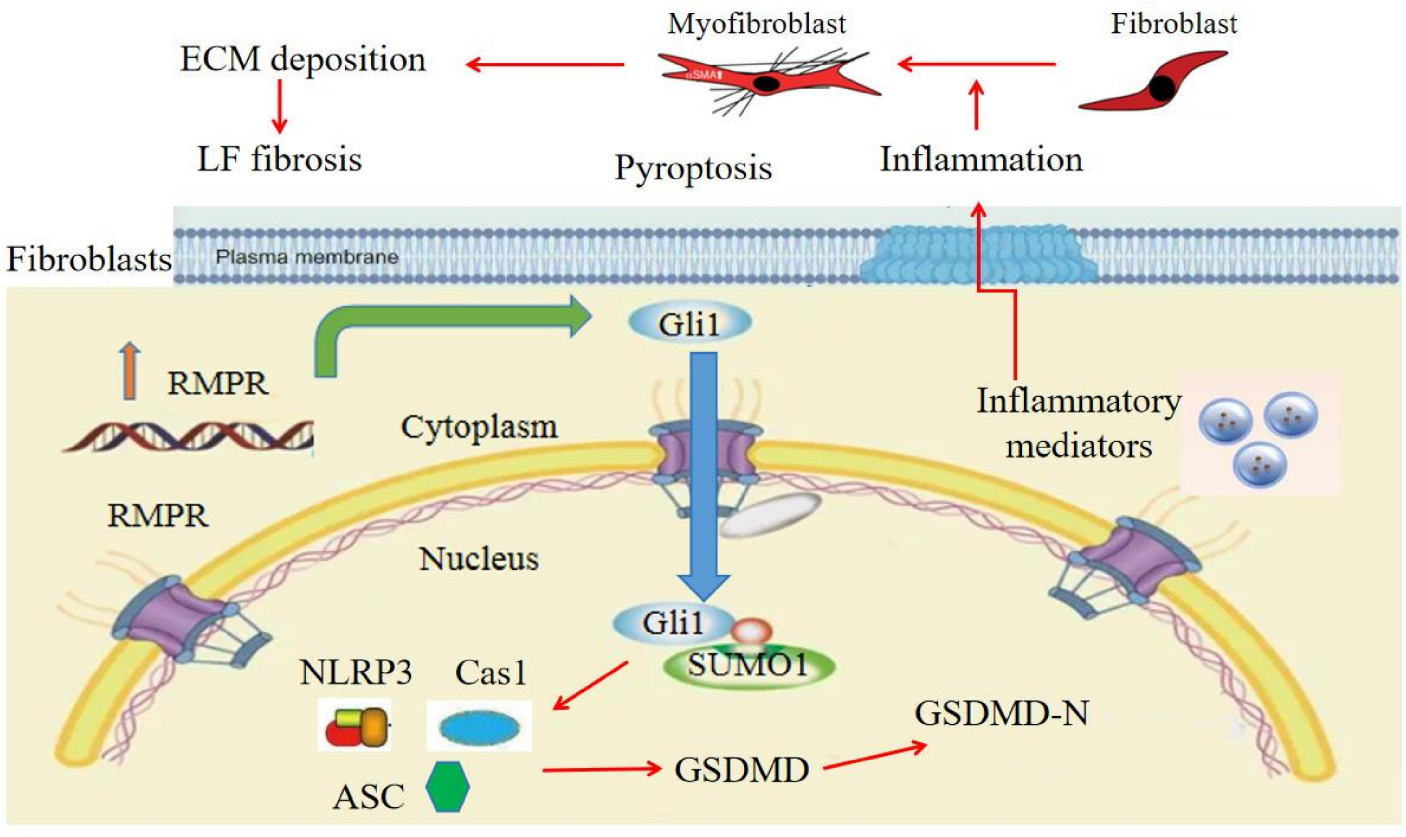
Proposed model for RMRP/Hedgehog-Gli1 pathway in LF fibrosis. RMRP activates Hedgehog-Gli1 pathway in LF cells, which facilitates SUMOlyation and nuclear translocation of Gli1, induces LF cell pyroptosis, and promotes local inflammatory response, ultimately resulting in collagen accumulation and LF fibrosis.

Collectively, the present study validates the significant role of the RMRP/Hedgehog-Gli1/pyroptosis profibrotic axis in LF hypertrophy and fibrosis and contributes to a better understanding of the molecular mechanisms underlying in LF hypertrophy (**Figure 6**). It is indicated that targeting RMRP may be a novel treatment strategy for addressing LF hypertrophy and may serve as a potential bioactive agent to mitigate the advancement of LF fibrosis in the future.

## Data availability statement

The datasets presented in this study can be found in online repositories. The names of the repository/repositories and accession number(s) can be found below: PRJNA1145660.

## Ethics statement

The studies involving humans were approved by Ethics Committee of Nanjing medical University. The studies were conducted in accordance with the local legislation and institutional requirements. The participants provided their written informed consent to participate in this study. The animal study was approved by Animal Care and Use Ethics Committee of Nanjing medical University. The study was conducted in accordance with the local legislation and institutional requirements. Written informed consent was obtained from the individual(s) for the publication of any potentially identifiable images or data included in this article.

## Author contributions

XY: Writing – original draft, Validation, Resources, Methodology, Investigation, Formal analysis, Data curation. TL: Writing – original draft, Supervision, Software, Methodology, Investigation, Formal analysis, Data curation. RZ: Writing – original draft, Validation, Supervision, Software, Methodology, Formal analysis, Data curation. QM: Writing – review & editing, Resources, Methodology, Formal analysis, Data curation, Conceptualization. CS: Writing – review & editing, Validation, Resources, Methodology, Investigation, Funding acquisition, Data curation, Conceptualization.
